# Lower leg muscle strengthening does not redistribute plantar load in diabetic polyneuropathy: a randomised controlled trial

**DOI:** 10.1186/1757-1146-6-41

**Published:** 2013-10-18

**Authors:** Tom Melai, Nicolaas C Schaper, T Herman IJzerman, Ton LH de Lange, Paul JB Willems, Valéria Lima Passos, Aloysius G Lieverse, Kenneth Meijer, Hans HCM Savelberg

**Affiliations:** 1 Department of Health Innovation & Technology, Institute of Allied Health Professions, Fontys University of Applied Sciences, Eindhoven, The Netherlands; 2 Department of Human Movement Sciences, NUTRIM School for Nutrition, Toxicology and Metabolism, Maastricht University Medical Centre, Maastricht, The Netherlands; 3 Department of Internal Medicine, CAPHRI Institute Maastricht, Maastricht University Medical Centre, Maastricht, The Netherlands; 4 ADL Biomechanical Consultancy, Rosmalen, The Netherlands; 5 Department Methodology & Statistics, Maastricht University Medical Centre, Maastricht, The Netherlands; 6 Department of Internal Medicine, Maxima Medical Centre, Eindhoven, The Netherlands

**Keywords:** Diabetes, Foot, Kinetics, Exercise, Physical therapy

## Abstract

**Background:**

Higher plantar pressures play an important role in the development of plantar foot ulceration in diabetic polyneuropathy and earlier studies suggest that higher pressures under the forefoot may be related to a decrease in lower leg muscle strength. Therefore, in this randomised controlled trial we evaluated whether lower-extremity strength training can reduce plantar pressures in diabetic polyneuropathy.

**Methods:**

This study was embedded in an unblinded randomised controlled trial. Participants had diabetes and polyneuropathy and were randomly assigned to the intervention group (n = 48) receiving strength training during 24 weeks, or the control group (n = 46) receiving no intervention. Plantar pressures were measured in both groups at 0, 12, 24 and 52 weeks. A random intercept model was applied to evaluate the effects of the intervention on peak pressures and pressure–time-integrals, displacement of center-of-pressure and the forefoot to rearfoot pressure–time-integral-ratio.

**Results:**

Plantar pressure patterns were not affected by the strength training. In both the intervention and control groups the peak pressure and the pressure–time-integral under the forefoot increased by 55.7 kPa (95% CI: 14.7, 96.8) and 2.0 kPa.s (95% CI: 0.9, 3.2) over 52 weeks, respectively. Both groups experienced a high number of drop-outs, mainly due to deterioration of health status and lower-extremity disabilities.

**Conclusions:**

Plantar pressures under the forefoot increase progressively over time in people with diabetic polyneuropathy, but in this study were not affected by strength training. Future intervention studies should take this increase of plantar pressure into account and alternative interventions should be developed to reduce the progressive lower extremity problems in these patients.

**Trial registration:**

This study was embedded in a clinical trial with trial number NCT00759265.

## Background

Diabetic polyneuropathy (DPN) is an important risk factor for elevated plantar pressures and consequently the development of plantar foot ulceration [1,2]. A foot ulcer is a feared complication of diabetes resulting in a high burden of disease for both the patient and the health care system. Diabetes is the leading cause of a lower extremity amputation in the world and more than 80% of these amputations are preceded by a foot ulcer [3]. Although major improvements have been made in ulcer treatment, the results of interventions that aim to prevent these ulcers are still disappointing [4] and new preventive strategies are needed.

In patients with DPN, especially the region of the forefoot is at higher risk due to deformities and altered gait patterns; higher loading of these areas has been associated with the development of plantar ulceration [2,5]. More specifically, Caselli et al. [2] found that in DPN there was a higher forefoot to rear foot ratio (F/R-ratio) and concluded that this ratio could help to predict the occurrence of ulceration. Currently, most preventive strategies aim to reduce the abnormal loading of the foot in patients at risk by prescribing offloading footwear and aids like shoe inlays [6]. However, these interventions do not correct the underlying pathology and mixed results have been obtained [7].

Several authors suggest that muscle weakness, limited joint mobility, delayed muscle activation and impaired sensory-mechanical feedback contribute to higher plantar pressures in the forefoot [8-10]. In a previous study [11], we found that DPN patients had an impaired ability to brake the forward momentum of the body just after heel strike, leading to a faster roll-off of the foot and consequently a higher loading of the forefoot. We observed close correlations between the plantar flexion moment at the first part of the stance phase, the rate of forward progression of the center-of-pressure and loading of the forefoot [12]. These results suggest that the decreased muscle strength due to DPN [13,14] can contribute to adverse plantar pressures. Therefore, we hypothesised that an intervention aimed at increasing lower limb muscle strength could enable participants to better counteract the forward momentum of the body during gait, thereby diminishing excessive forefoot loading. The aim of the current study was to evaluate the effects of lower extremity strength training on plantar pressures in DPN.

## Methods

### Design overview

The current study was embedded in a randomised controlled trial that was carried out in two institutes (trial registration: NCT00759265). The primary outcome measure of the trial was muscle function and the main secondary outcome was mobility. Hence, sample size of the original trail was determined based on the expected distribution and effects of exercise on muscle function, not on plantar pressures. Based on scarce literature [15,16] the sample size of the trial was determined at 50 participants with DPN for both the intervention and control group. The sample size of the current study was based on this sample size determination and therefore can be considered a convenience sample. Due to practical reasons it was not possible for research staff and participants to be blinded for treatment allocation. There is, to the best of our knowledge, little information on the effect of muscle strength training on plantar pressures in patients with DPN. Plantar pressure parameters were therefore included as secondary endpoints in the trial and will be reported in the current study. Outcome measures on muscle strength and mobility will be reported elsewhere.

### Setting and participants

Participants were recruited from five diabetes outpatient clinics (one university and four regional hospitals) in the southern region of the Netherlands between December 2006 and November 2010. To be included, participants had to be above 50 years of age and had to be able to walk 6 minutes without walking aids. Participants were excluded if diagnosed with severe cardiac disease (New York Heart Association classification ≥ 3), renal dysfunction (creatinin > 180 μmol/l), intermittent claudication, neurological disorders other than DPN, rheumatoid arthritis, amputations, prior or current foot ulceration or were thought to be at risk of falling as assessed by the research staff. Participants were also excluded if the presence of severe osteoarthritis or foot deformities interfered with their gait.

The diagnosis of DPN was made in 94 participants, scoring at least 4 points out of the maximal 33 during a standardised clinical neurological examination, which included reflexes, light touch, vibration, position sense, pinprick and lower extremity muscle strength testing [17]. The protocol was approved by the medical ethical committee of the hospitals involved and all participants signed informed consent.

Participants were tested in one of the two participating institutes. At both institutes a similar set-up was present with a seven meter wooden walkway with an embedded pressure platform (at the first institute: emed®-x, 100 Hz, 4 sensors/cm^2^, range 0–1270 kPa, Novel Inc., Munich, Germany. At the other institute: emed®-at, 50 Hz, 2 sensors/cm^2^, range 0–1200 kPa, Novel Inc., Munich, Germany). Each participant was measured at the same institute and with the same platform throughout the study. Gait velocity was measured using custom-made infrared detection gates placed two meters apart over the platform.

### Randomisation and intervention

**Figure 1:**
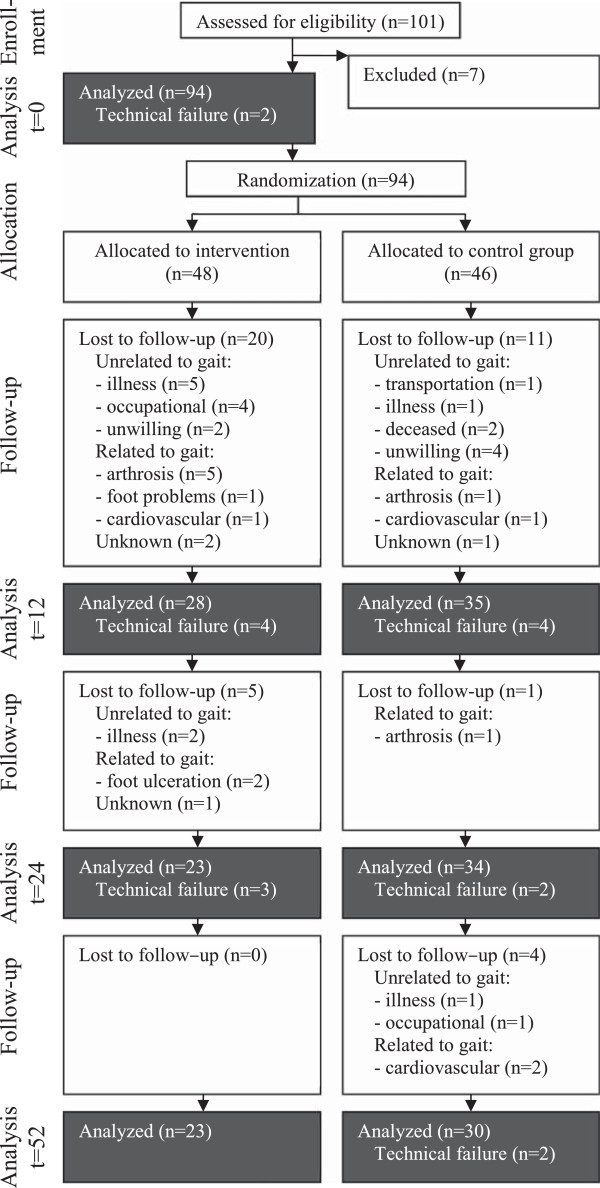
**Design, number of analyzed subjects and reasons for attrition.** The numbers represent the subjects analyzed at different times of measurement (at t = 0, 12, 24 and 52 weeks), including attrition due to technical failure. The lost to follow-up includes the reason for drop-out, which was classified as related or unrelated to gait.

After baseline measurement, participants were randomised by research staff using the envelope method (opaque, sealed envelopes) stratified over five groups (two at the first institute and three the other), to either the intervention group receiving physical therapeutic training or to the control group receiving no training (Figure 1). Both groups were free to perform any additional physical activity outside the trial.

The participants assigned to the intervention received weekly plenary training for 24 weeks. The intervention was developed by a multidisciplinary team, aiming to improve muscle strength of the lower limb. Every session was guided by a physical therapist under supervision of a member of the research team. During the first 12 weeks (part 1) the focus was on the lower leg, training of the dorsal and plantar flexors, and lower leg coordination. During the next 12 weeks (part 2) the entire lower extremity was trained.

Each training session consisted of four sections. The first section provided a warm up by simple exercises performed during gait. The second section included lower extremity strength training, gradually increasing from low to moderate intensity at 40 to 60% of one repeated maximum, and small, mostly seated, coordinative tasks. The materials used to build up resistance during these exercises were elastic bands, weight vests and ankle weights. The third section included functional gait tasks in terms of a challenging gait track with obstacles resembling activities of daily life. Each training lasted one and a half hours and was concluded with a selection of various interactive tailored games. In addition to these plenary sessions, participants were provided with an exercise manual and were asked to carry on the exercises of the second section, two times a week at home, without supervision or monitoring. More detailed information on the training program has been made available (please see Additional file 1: training schedule).

### Measurement protocol

During measurements, all participants wore shorts, walked barefoot and were allowed to familiarise themselves with the test settings of the plantar pressure measurements. Testing was performed at two gait velocities. At first the participants were asked to walk over the test track at their own preferred gait velocity, which was allowed to vary each trial. Subsequently, they had to complete the test at a standardised imposed gait velocity within a range of 1.1 to 1.3 m/s, as this resembles the preferred gait velocity for this age group [11]. At both velocities they had to complete preferably five, but at least three successful trials. A trial was successful if the participants did not alter their gait pattern to target the platform, which was assessed by the research staff. For practical reasons and comparability over time, only data was collected for the right foot.

Measurements were performed at baseline (t = 0), after the first part of the intervention, at 12 weeks (t = 12), after the second part of the intervention, at 24 weeks (t = 24) and at follow-up, after 52 weeks (t = 52). If a participant did not wish to participate anymore in the study, they were asked to report the reason why. To monitor whether there was a possible relation with the intervention, the reasons given were classified as either gait-related or not gait-related (Figure 1).

### Data analysis

The effect of the training program on plantar pressures was considered the primary outcome of the current study. Plantar pressure data were masked in Novel Database Medical (13.3.42, Germany 2007) using the Novel 10 mask division (area 1 = heel, area 2 = mid foot, areas 3-7 = metatarsal region, area 8 = hallux, area 9 = second toe and area 10 = smaller toes) [18]. If there were inconsistencies in the automatic masking procedure, due to, for example, dragging of the hallux over the pressure platform, trials were excluded. For each trial the peak pressure, the force-time-integral (FTI) and the contact area per foot region were calculated. Using Matlab (R2007b, USA 2007), the pressure–time-integral (PTI) was calculated as the quotient of the FTI and the contact area for each mask [18]. The PTI of the forefoot was calculated as a quotient of the summation of FTI for area 3 to 10 and the total contact area of these regions. Peak pressure of the forefoot was set as the highest peak pressure in area 3 to 10. The forefoot to rearfoot ratio (F/R-ratio) was calculated by taking the quotient of the PTI of the forefoot and the heel. The displacement rate of center-of-pressure (COP) was operationalised as the percentage of the stance phase needed for the COP to reach the forefoot (area 3–10): this variable was abbreviated as tCOP. A minimum of three correct measurements were averaged for each participant per measurement (t = 0, 12, 24 or 52).

Biochemical analysis was performed to monitor overnight fasting blood glucose, glycated hemoglobin (HbA1c) and creatinin, at t = 0, 24 and 52.

### Statistical analysis

All data are expressed as mean with 95% confidence intervals (CI). The effect of the training program on the primary outcome was determined on an intention to treat analysis. As participants participated in a training program with two components and were subsequently followed for 6 months, multiple measurements had to be performed over time. Given the hierarchical structure of the data, with repeated measurements nested within participants, a random intercept model was fitted to evaluate average changes in plantar pressure variables over time for the intervention and control group, adjusting simultaneously for covariates. This model has the advantage of using all available observations for model estimation, inclusively those of participants entered in the model until drop-out, and not only the complete cases like the classical repeated measures ANOVA.

Ideally both preferred and imposed gait velocity should be evaluated simultaneously. However, not all participants were able to complete the imposed gait velocity, so this analysis was performed separately for the two gait velocities.

The time point at follow-up (t = 52) was used as a reference for comparisons involving temporal changes. Group, gender, body mass and age were entered as fixed factors. Participants made up the random factor. The interaction between group and time was considered in order to test whether temporal changes of the outcomes over time depended on the group, with a significance level of 5%. Estimates were determined by a restricted maximum likelihood method. For interaction terms, whose *p*-values were < 0.1, additional Likelihood Ratio (LR) tests were conducted (at 5% significance level). If non-significant, they were left out of the final model. All other fixed factors remained in the model, irrespective of significance.

It should be noted that in these multivariable models, the fixed effect parameters are unbiased, if attrition was non-informative, i.e. missingness conditioned on each covariate occurred at random. However, the pattern of missingness within groups, for instance, indicates that this condition may not hold. Therefore, results should be interpreted with caution (as will be discussed further on).

All data were analysed using SPSS 15.0 for Windows.

## Results

### Participant characteristics

Participants were overall obese with a good to moderate glycemic control (Table 1). The majority was male and had moderate DPN (clinical neurological examination score). Participants in the control group were on average three years younger. All other characteristics did not differ significantly between groups. At 52 weeks follow-up, both groups had the same body mass as at baseline, but increased their preferred gait velocity. At baseline, 21 participants (12 in the intervention group and 9 in the control group) were not able to reach the imposed gait velocity due to their health status. During the consecutive measurements, the numbers of participants that did not reach this faster gait velocity were 1, 2 and 4 respectively for the intervention group and 6, 5 and 6 respectively for the control group.

**Table 1:** Participant characteristics at baseline (values are means and SDs unless otherwise indicated)

	**Intervention**	**Control**
Age (years)	68.4 (7.3)	65.2 (7.2)*
Sex (M/F)	40/8	36/10
Height (m)	1.74 (0.08)	1.74 (0.08)
Body Mass (kg)	92.2 (20.1)	94.2 (19.1)
BMI (kg/m^2^)	30.4 (6.2)	30.9 (4.9)
CNE score	12.9 (4.9)	12.8 (5.3)
HbA1c (mmol/mol)	56 (12)	56 (12)
Glucose (mmol/L)	8.8 (2.6)	8.2 (2.0)
Creatinine (μmol/L)	100.9 (35.1)	94.8 (23.5)

Notes: Age determined at onset of study. Abbreviations: *CNE* Clinical Neurological Examination; *M* Male; *F* Female * Significant difference between intervention and control group (*p* < 0.05).

Both groups experienced a high number of drop-outs during the first 12 weeks (Figure 1). In most cases, attrition was caused by deterioration of health status prohibiting the participants to further participate in the measurements or intervention. The reasons for drop-out as reported by participants are specified in Figure 1. Drop-out was particularly high for the intervention group in the first 12 weeks (41.7% in the intervention group and 23.9% in the control group, *p* = 0.067). The reason for stopping with the intervention was gait-related in 8 of the 20 patients who dropped-out. In the control group, 11 participants dropped-out; 2 because of gait-related reasons. Throughout the full study, 11 of the 25 participants (44%) stopped with the intervention because of gait-related problems and in the control group this number was 5 out of the 16 (31.3%).

### Effect of training on plantar loading

No significant effect of the intervention on the plantar pressures characteristics (peak pressures, PTI, tCOP and F/R-ratio) was observed (LR p-values >0.1). As the interaction effect (time*group) was not significant for any of the variables it was left out of subsequent models. Therefore, the random intercept model was constructed of age, gender (male versus female), body mass, group (intervention versus control) and time of measurement (t = 0, 12 and 24, versus 52 weeks).

### Effect of time on plantar loading

**Figure 2:**
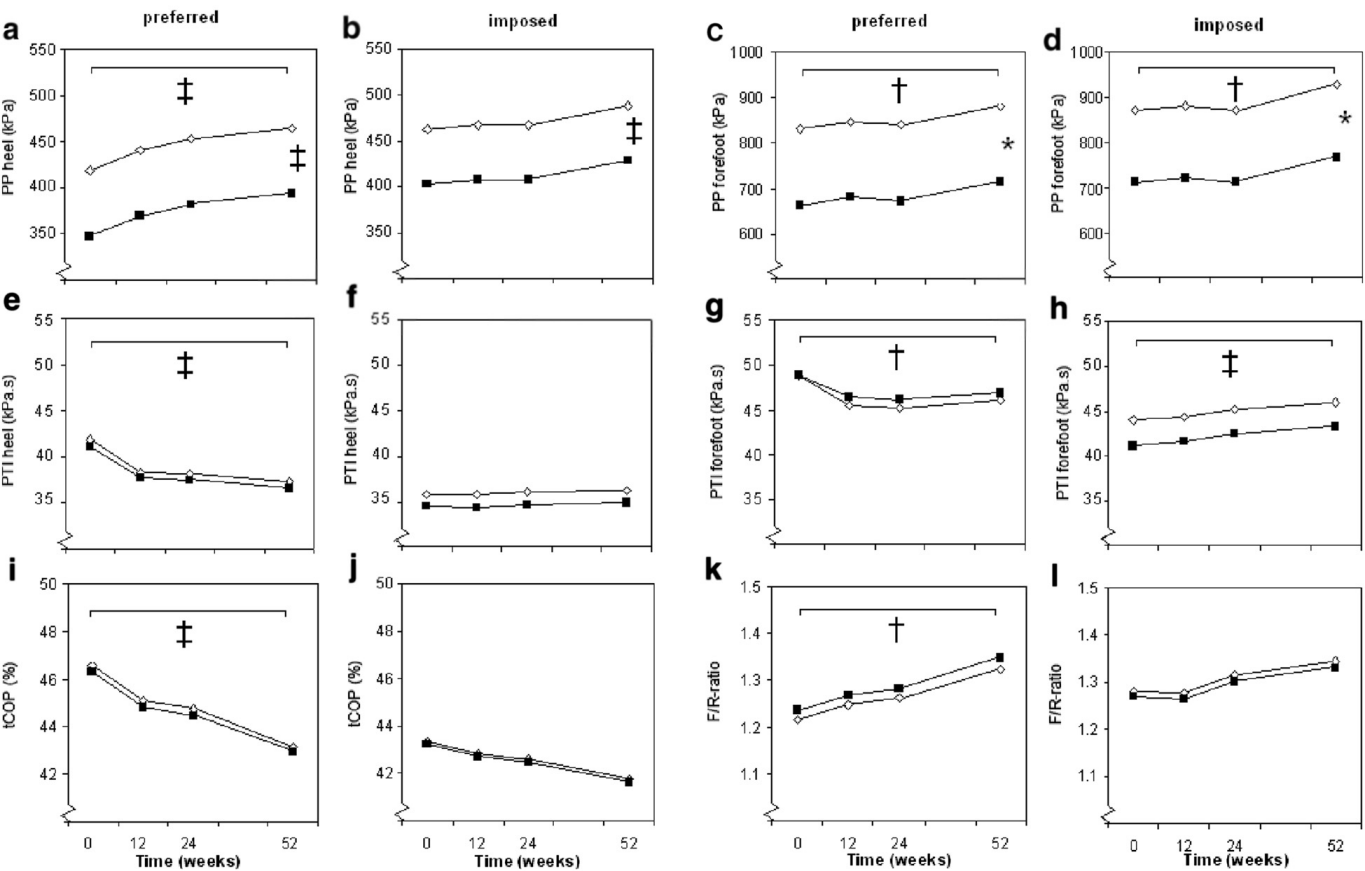
**Estimated mean values over time for intervention (in black) and control groups (in white). (a-l).** Represented outcome parameters are according to the final random intercept models (Table 2). Graphs have broken y-axes to improve readability. PP is peak pressure; PTI is pressure–time-integral; tCOP is center-of-pressure expressed as percentage of the stance phase for it to enter the forefoot; F/R-ratio is the forefoot to rearfoot PTI ratio. Significant time effects over 52 weeks and group differences are marked with: * = *p* ≤ 0.05, † = *p* ≤ 0.01, ‡ = *p* ≤ 0.001.

Time effects were present for almost all plantar pressure parameters, regardless of the group. At the preferred gait velocity, the peak pressure under the heel increased over 52 weeks by 45.8 kPa (95% CI: 25.3, 66.2; *p* ≤ 0.001; Table 2 and Figure 2a). At the preferred and imposed gait velocity, the peak pressure under the forefoot increased in both groups by 52.8 (95% CI: 18.0, 87.5) and 55.7 (95% CI: 14.7, 96.8) kPa, respectively (*p* ≤ 0.01; Table 2 and Figure 2c-d). At the *preferred gait velocity,* the PTI of the heel and forefoot decreased in both groups by 4.5 (95% CI: 2.6, 6.7) and 2.0 (95% CI: 0.4, 3.5) kPa.s, respectively (*p* ≤ 0.01; Table 2 and Figure 2e and g). In contrast, at the *imposed gait velocity* the PTI increased under the forefoot in both groups by 2.0 kPa.s (95% CI: 0.9, 3.2; *p* ≤ 0.01; Table 2 and Figure 2h). Moreover, tCOP decreased in both groups during the preferred gait velocity by 3.4% (95% CI: 1.92, 4.90; *p* ≤ 0.01) as shown in the Table 2 and Figure 2i and j, indicating a faster forward transfer of COP. Also F/R-ratio increased over time for the preferred gait velocity by 0.11 (95% CI: 0.18, 0.38; Table 2 and Figure 2k; *p* ≤ 0.01).

**Table 2:** **(a-l): The determinants and their effects on plantar pressures in diabetic patients with polyneuropathy depicted as **$\hat{\beta }$** (CI 95)**

	**Preferred**	**Imposed**	**Preferred**	**Imposed**
	**a. PP heel (kPa)**	**b. PP heel (kPa)**	**c. PP forefoot (kPa.s)**	**d. PP forefoot (kPa.s)**
Intercept	358.6 (120.0, 597.1)†	149.2 (−110.0, 408.3)	955.1 (396.6, 1513.5)‡	706.4 (84.5, 1328.3)*
Age	−2.3 (−5.1, .6)	0.1 (−3.2, 3.4)	−4.6 (−11.4, 2.1)	−1.4 (−9.2, 6.4)
Body mass	2.1 (.8, 3.4)†	3.0 (1.6, 4.3)‡	−1.1 (−2.0, 4.1)	2.1 (−1.2, 5.3)
Group (IG vs CG)	72.0 (33.7, 110.3)‡	60.4 (20.9, 99.8)†	168 (82.8, 253.5)*	162.4 (70.9, 253.9)*
Time t = 0 vs 52	−45.8 (−66.2, -25.3)‡	−25.6 (−45.9, -5.3)	−52.8 (−87.5, -18.0)†	−55.7 (−96.8, -14.7)†
Time t = 12 vs 52	−23.7 (−45.1, -2.5)*	−20.5 (−41.0, 0.1)	−36.0 (−71.5, -.3)*	−46.4 (−87.7, -5.2)*
Time t = 24 vs 52	−11.7 (−33.3, 9.9)	−20.5 (−41.4, 0.4)	−42.4 (−78.4, -6.4)*	−55.4 (−97.4, -13.3)*
$\sigma_{i}^{2}$	296.7 (237.7, 370.3)‡	218.0 (169.9, 279.6)‡	821.1 (655.7, 1028.2)‡	871.6 (676.3, 1123.3)‡
$\sigma_{b_{0}}^{2}$	868.2 (611.9, 1232.0)‡	897.9 (623.8, 1292.4)‡	5858.9 (4225.9, 8122.9)‡	5848.5 (4084.7, 8373.8)‡
	**e. PTI heel (kPa.s)**	**f. PTI heel (kPa.s)**	**g. PTI forefoot (kPa.s)**	**h. PTI forefoot (kPa.s)**
Intercept	3.6 (−21.8, 29.0)	31.9 (14.3, 49.3)‡	8.2 (−8.0, 24.4)	25.3 (10.0, 40.6)†
Age	.1 (−.2, .4)	-.3 (−.6, -.1)†	.2 (−.0, 0.4)	-.8 (−.3, 1.2)
Body mass	.3 (.1, .4)‡	.3 (.2, .4)‡	.3 (.2, .4)‡	.2 (.2, .3)‡
Group (IG vs CG)	.7 (−3.3, 4.8)	1.3 (−1.5, 4.0)	-.8 (−3.5, 1.8)	2.8 (.5, 5.1)
Time t = 0 vs 52	4.6 (2.6, 6.7)‡	-.3 (−2.0, 1.4)	2.0 (.4, 3.5)†	−2.0 (−3.2, -0.9)‡
Time t = 12 vs 52	1.1 (−1.0, 3.3)	-.4 (−2.2, 1.3)	-.6 (−2.2, 1.1)	−1.7 (−2.9, -.5)†
Time t = 24 vs 52	.9 (−1.3, 3.1)	-.1 (−1.9, 1.6)	-.8 (−2.5, .8)	−0.8 (−1.9, .4)
$\sigma_{i}^{2}$	3.0 (2.4, 3.7)‡	1.6 (1.2, 2.0)‡	1.7 ( 1.4, 2.2)‡	.7 (.5, .9)‡
$\sigma_{b_{0}}^{2}$	10.3 (7.2, 15.6)‡	3.5 (2.3, 5.2)‡	3.7 (2.5, 5.5)‡	3.2 (2.2, 4.7)‡
	**i. tCOP (%)**	**j. tCOP (%)**	**k. F/R-ratio**	**l. F/R-ratio**
Intercept	33.16 (15.05, 51.28)‡	51.58 (35.05, 68.11)‡	1.31 (0.52, 2.09)†	.93 (.07, 1.80)*
Age	.03 (−.19, .25)	-.26 (−.47, -.06)†	.00 (−.01,.01)	.01 (−.00, 0.02)
Body mass	.09 (−.01, 0.19)	0.09 (0.01, 0.18)*	-.00 (−.01, .00)	-.00 (−.01, .01)
Group (IG vs CG)	.26 (−2.64, 3.15)	.12 (−2.48, 2.72)	-.02 (−.15, .11)	.01 (−.12, .15)
Time t = 0 vs 52	3.41 (1.92, 4.90)‡	1.60 (−0.03, 3.22)	-.11 (−.18, -.38)†	-.06 (−.14, .021)
Time t = 12 vs 52	1.93 (.39, 3.48)*	1.07 (−.60, 2.74)	-.08 (−.15, -.02)*	-.07 (−.15, .019)
Time t = 24 vs 52	1.59 (.03, 3.16)*	.83 (−.87, 2.53)	-.06 (−.14, .01)	-.03 (−.11, .06)
$\sigma_{i}^{2}$	15.64 (12.53, 19.51)‡	14.48 (11.31, 18.54)‡	0.04 (0.03, 0.05)‡ .000	.04 (.03, .05)‡
$\sigma_{b_{0}}^{2}$	51.49 (36.53, 72.58)‡	30.95 (20.80, 46.04)‡	0.09 (0.06, 0.13)‡ .000	.09 (.06, .13)‡

Notes: Measurements were obtained for the intervention and control group (Group) at different time points (Time) and at two gait velocities, preferred and imposed (see text). The main estimated fixed (and random) effect parameters of the random intercept models are shown. β (95% CI) represent average changes in the outcome variables per unit increase for the continuous explanatory variables. For categorical variables, the estimates represent average changes with respect to the reference (control group, females, or follow-up measurement at t = 52 weeks). Effects of time are negative if baseline measurements were lower than during follow-up. Age was determined at onset of the study. Abbreviations and symbols: *IG* intervention group; *CG* control group; *PP* peak pressure; * = p ≤ 0.05, † = *p* ≤ 0.01, ‡ = *p* ≤ 0.001.

### Overall group differences

At all times of measurement, there were no differences between the intervention and control group for PTI, tCOP and F/R-ratio, but the peak pressures under the heel and forefoot were significantly higher for the intervention group for both the preferred and imposed gait velocity (*p* ≤ 0.01, Table 2 and Figure 2a-d).

### Effects of covariates

For every extra year of age, PTI of the heel was lower (*p* ≤ 0.01, Table 2f) and the COP reached the forefoot earlier (*p* ≤ 0.01, Table 2j) at the imposed gait velocity. For both the preferred and imposed gait velocity, an increase in body mass of one kilogram was associated with increased loading of the heel in terms of peak pressure and PTI (*p* ≤ 0.01, Table 2a-d). The same was observed for the PTI under the forefoot for both gait velocities (*p* ≤ 0.01, Table 2g and h). Gender did not have a significant effect on any of the outcome parameters and was for the sake of clarity omitted from Table 2.

## Discussion

In the current study we tested the effect of a gait and strength training program on plantar pressure distribution in DPN. Our main finding was that the exercise program did not affect plantar pressure patterns. As we did not observe a difference between the intervention and control groups, we could combine both arms in a prospective observational study, in which we observed an increase in forefoot loading over time in participants with DPN, during the individually preferred and standardised imposed gait velocity.

As far as we know, only one other study has examined the effects of exercise training on plantar pressures; in postmenopausal women a reduction in peak plantar pressures at preferred gait velocity was observed after 12 months training but the underlying mechanism of this reduction was not provided [19]. It is unclear why our intervention did not affect plantar pressure patterns. The high drop-out rate might have influenced the outcome, but the intervention did increase maximal plantar flexion strength, although maximal dorsiflexion strength was not affected (to be reported elsewhere: IJzerman et al. Unpublished data). The lack of effect on dorsiflexion strength and plantar pressures could be explained by either insufficient intensity of the training or impaired trainability due to the underlying neuropathy of DPN patients. Alternatively the increased forefoot loading in DPN may not originate from muscle weakness per se, but rather from abnormalities in other factors associated with joint motion, motor control, feedback from muscle spindles, rate of force development and sensory nerve function of the skin [10,20-22].

Our trial resulted in some important findings that should be taken into account in future research exploring the effects of other or similar interventions on plantar pressures in patients with DPN. Firstly, it is possible that we did not observe an effect of our intervention, due to a large between-participants variability. In our research we included patients with different types of diabetic polyneuropathy, several mild comorbidities and several levels of motivation. To see which intervention is most suitable for the patients being helped, future research needs to focus on strictly defined specific patient groups before looking at general benefits of an intervention in a randomised controlled trial.

Secondly, we observed an increase in forefoot plantar pressure over time in both the intervention and control group. These changes in pressure distribution differed between the preferred and imposed gait velocity conditions. During the preferred gait velocity, peak pressure under the heel and forefoot increased and the PTI under the heel and forefoot decreased over time. The COP reached the forefoot earlier and F/R-ratio was higher after one year. These changes over time are probably related to the increased gait velocity in our participants when participants could determine their own gait velocity. An increased gait velocity would lead to shorter plantar loading and therefore a lower PTI. Although all participants were familiarised with the experimental conditions, it is possible that this increased gait velocity was caused by habituation to the experimental settings.

A different pattern of the plantar pressures over time was seen when gait velocity was kept constant. During the standardised imposed gait velocity, both the peak pressure and PTI under the forefoot increased over time in participants with DPN. Our data clearly indicate that gait velocity should be taken into account in future studies and when studying mechanisms of elevated plantar pressures or the effect of interventions, gait velocity should be standardised in at least one set of measurements.

Earlier studies also reported an increase of plantar pressures over time [1,2], but only a few provided quantitative information. It has been reported that during a mean follow-up of 34 months mean plantar pressure increased by 65 kPa in people with diabetes type 2 unspecified for neuropathy status [23]. In participants with type 1 or type 2 diabetes and DPN plantar pressure increased by 255 kPa over a mean follow-up of 30 months [24]. Both studies did not report whether they controlled or monitored gait velocity. In this study we observed in participants with type 2 diabetes and DPN an increase after 12 months under the forefoot of 2.0 kPa.s for the PTI and 55.4 kPa for the peak pressure (Table 2h and d), during the imposed gait velocity. High plantar pressures are an important risk factor for foot ulceration and in one prospective study, patients who developed an ulcer during the 2-year observation period had a mean baseline peak pressure of 955 ± 264 kPa, while non-ulcerated participants had a peak pressure of 851 ± 273 kPa (p < 0.001) [25]. The difference of 100 kPa between the ulcerated and non-ulcerated participants was less than 2 times the increase of pressure we observed in one year. Therefore, the risk for ulceration in patients with DPN seems to increase rapidly over time.

Other information that could be useful for future research can be obtained from the limitations of the current study. A major limitation was the high drop-out rate, especially during the first 12 weeks of the training program. The application of a random intercept model circumvented, at least partly, the problem of missing data irrespective of the differential attrition rate between the groups. Via this modeling approach, the estimated mean values of the outcome parameters, contingent on the model covariates, can be considered unbiased if the pattern of missingness within each experimental group were at random. In the intervention group more participants dropped out because of gait-related problems, which might have introduced bias in the final parameter estimates. Progression of polyneuropathy often leads to progression of morbidity [26] and an increase in plantar loading [2]. Therefore, it is conceivable that participants that refrained from the study had higher plantar pressures than the participants that completed the study.

The higher drop-out rate could not be directly attributed to overuse injuries, but we cannot rule out the possibility that the training contributed to deterioration of the health status of participants that dropped out. Gait-related problems were a major reason for drop-out in one third of the participants who withdrew from the study and in addition, the staff had the impression that lack of motivation also played an important role in several patients. The motivation to exercise is probably suboptimal in many people with diabetes, as people that are less motivated to exercise throughout their lives have a higher chance to develop diabetes and its complications [27,28]. A high drop-out rate was also a major problem in other long term exercise programs in patients with diabetes (e.g. a drop-out rate of 60% was observed during brisk walking programs) [19,29,30]. To prevent participant drop-out in future interventions, more emphasis should be placed on the prevention of gait-related problems, on improving selection criteria with respect to health status and motivation, and on keeping participants motivated. Finally, the high drop-out rate should be taken into account in power calculations for sample size estimations.

Another limitation of the current study is that the sample size was determined based on the expected distribution and effects of exercise on muscle function, not on plantar pressures. In addition, since no correction for multiple testing was applied, significant findings should be interpreted with caution. The study was set up with a single-blinded design, with outcome assessors being blinded for the allocation of participants to groups. However, as participants were not able to conceal their allocation, blinding of assessors could not be assured. Nevertheless, plantar loading was measured with an objective procedure and during the data analysis participant codes were used that could not be related to treatment allocation. Finally, it could be considered a limitation that we did not use identical pressure platforms at the two study sites, which in theory could have affected the outcome. Nevertheless, we expect the difference in platforms to be negligible, as the platforms were made by the same manufacturer, provide comparable data, and because our primary outcome was the change in plantar pressure.

## Conclusions

In conclusion, a lower extremity strength training program was not successful in reducing plantar loading of the forefoot for people with diabetic polyneuropathy. Forefoot loading increased in both the intervention and control group over time resulting in a progressive rise in the risk of foot ulceration. The results described in this article provide valuable data for the design of future mechanistic and intervention studies.

## Competing interests

The authors declare that they have no competing interests.

## Authors’ contributions

All authors were involved in the conception and design of this study. TM and THIJ carried out data acquisition and study site coordination. PJBW lead the data acquisition and set up analysis and data processing. VLP lead the statistical analysis and TM, NCS, HHCMS, TLHdL, carried out or participated in analysis and interpretation of the data. TM wrote the first draft of the manuscript, with the help of NCS, HHCMS and TLHdL. All authors read and approved the final version of the manuscript.

## Supplementary Material

Additional file 1 Training schedule. Click here for file
