# Supplementary material for: Lower leg muscle strengthening does not redistribute plantar load in diabetic polyneuropathy: a randomised controlled trial
Source: J Foot Ankle Res. 2013 Oct 18;6:41. doi: 10.1186/1757-1146-6-41 (PMC4015645; doi:10.1186/1757-1146-6-41)
Supplement: Additional file 1 — Training schedule. [file 1757-1146-6-41-S1.pdf]

Training schedule

The training period lasted 24 weeks, with 3 training sessions per week. Once a week there was a plenary training, consisting of four sections: a warm-up, strength and balance training, gait training and adjusted games. Apart from this plenary training the participants were asked to continue the strength training twice a week at home during 30 minutes.

Warm-up

The warm-up was the same every week, lasted for approximately 10 minutes and consisted of walking laps in the training hall at an individual pace. During walking various assignments were given:

- Stand still and stretch as far as you can: arms as high as possible over your head while standing on your toes;
- Stand still and 'roll down' starting with your head, vertebra by vertebra;
- Swing your arms and spin around;
- Walk on your toes:
- Walk on your heels;
- Walk with long steps;
- Walk with wide steps;
- Tap your heels against your buttocks;
- Pull up your knees;
- Shake loose your arms and legs.

Strength and balance training

The strength and balance training was performed sitting on a chair (during most exercises) with a duration of 25 minutes. The exercises were performed with constant resistance during 4 weeks. The first week started out with 3 sets of 4 repetitions. This was increased by 2 repetitions per week until 3 sets of 10 repetitions were performed in the fourth week. After the fourth and eight week the exercise resistance was increased and the participants started with 3 sets of 4 repetitions again, followed by the same schedule.

The schedule is schematically presented in a table.

Week	1/5/9	2/6/10	3/7/11	4/8/12
Number of series x repetitions	3x4	3x6	3x8	3x10

The strength and balance training consisted of two components. During the first 12 weeks of the training programme it was mainly aimed at the lower leg; the exercises were:

- Resisted plantar flexion. Participant standing up, if necessary supported by a chair. Heel raises with repeatedly standing on toes.
- Coordination of the lower leg muscles. Participant folds and unfolds a towel with feet while sitting on a chair.
- Resisted foot eversion. Participant sits, secures elastic band with predetermined resistance around balls of feet. Feet are placed at hip width on the heels, participant turns the right foot repeatedly from neutral position to eversion and back.
- Balance and stability exercises. Participants in twos, one performs the exercise, the other assists and offers support if necessary. Participant is standing behind a chair, feet at hip width, closes the eyes and stays standing, if possible without support. In

the course of the weeks the difficulty level is increased by standing on one leg and finally to standing on one leg with eyes closed.

- Balance and stability exercises. Participants in twos, one performs the exercise, the other assists and offers support if necessary. Participant is standing behind a chair, feet approximately at hip width, lifts feet off the floor and holds the knee in 90° flexion, closes eyes, if possible without taking support.
- Resisted dorsiflexion and plantar flexion. Participant sits, secures elastic band with predetermined resistance around balls of feet. Participant places heels on the floor, ankle in approximately 90°, the right foot moves toward plantar flexion and back, the left foot is kept still.
- Resisted dorsiflexion and plantar flexion of the foot. Participants in twos, sitting across from each other. One secures elastic band with predetermined resistance around the forefoot, while the other secures both ends under the foot. The first participant moves the foot repeatedly towards dorsiflexion and back to a 90° angle.

The second component took place during weeks 13 until 24 and was aimed at strengthening the entire leg; the exercises were:

- Strength training of the hip and knee extensors. Participant sitting with elastic band with predetermined resistance under the right foot, the two ends secured in his or her hands. Starting with the knee at the chest, the leg is repeatedly extended towards the floor and slowly back to the starting position.
- Strength training of the hip and knee extensors. Participant standing in front of a step bench or comparable bench. Participant steps with one foot on the bench followed by the other foot. Then both feet return to the starting position.
- Strength training of the hip and knee extensors. Participants squat starting from a standing position (move buttock backward to relieve the knees), as if they want to pick something up from the floor. Then back to a standing position.
- Strength training of the hip and knee extensors. Participants standing with chair approximately 1m in front of them. Participants make lunges forward, alternating right and left leg. Both legs are flexed, the knee of the hind leg in an angle of approximately 90°, with the knee moving towards the floor.
- Strength training of the hip and knee extensors. Participants sitting. Starting from this position each participant moves towards a full standing position, without using the arms/hands, and back to a sitting position. To ensure that the participants are actually sitting down, they must place a cone from the left side of the chair to the right side, or vice versa.
- Strength training of the hip adductors. Participant sitting with soft ball between the knees, the hands are kept on the back. Participant squeezes the ball empty between the knees, and slowly lets it inflate again.
- Strength training of the hip abductors. Participants sitting with elastic band with predetermined resistance is knotted close-fittingly around the knees. The feet are approximately at hip width on the floor. The knees are alternately pulled away from each other and slowly back towards each other.
- Strength training of the knee flexors. Participants sitting with ball under the heel. Both hands are kept on the back, the ball is pressed flat with the foot, then slowly allowed to inflate.

Gait training

A gait training was offered during thirty minutes in the form of an exercise track, resulting in coordination training on a functional level.

- Diagonal ramp: ramp is diagonal to the walking direction (imitates slopes).
- Balance beam: path of 6m long, 15cm broad, two bends at right angles. The feet must be placed entirely on the strip while walking over the path.
- Walking on various surfaces (soft mattress, deep-pile carpet, grass, sand, rubber mats, foam rubber).
- Climbing a ramp, turn and back down: 2-3 benches attached to the wall bars next to each other at an obtuse angle (supervised).
- Cones are placed around the ends of a bench: participant walks in between the bench and the cones (imitates small passage, e.g. around a coffee table).
- Picking up balls from a box on the ground, bring it to another box.
- Stepping over a bench.
- Stepping stones: participant walks the track with long paces.
- Slalom: 6 cones with a stick standing in them, walk between them without touching the sticks or cones.
- Pirouette: on a disk with a diameter of 40cm, turn 360 degrees without moving off the surface of the disk.
- Sitting down and standing up without arm support, chair is 35cm high. To ensure that the participants are actually sitting down, they must place a cone from the left side of the chair to the right side while sitting down, and vice versa.
- Walking while pushing a ball continuously with the foot, without losing control of the ball.

Games

Each plenary training session was concluded with fifteen minutes of interactive games, such as volleyball, badminton or basketball. Because of the decreased load tolerance of the participants the games were adjusted, e.g. by replacing the ball with a balloon or beach ball.
